# Rhizosphere microbiomes in drought-tolerant and drought-sensitive bermudagrass genotypes: root exudate association

**DOI:** 10.3389/fmicb.2026.1868900

**Published:** 2026-07-23

**Authors:** Sayada Momotaz Akther, Daniel Krakko, Wei Shi

**Affiliations:** 1 Department of Crop and Soil Sciences, North Carolina State University, Raleigh, NC, United States; 2 The Molecular Education, Technology, and Research Innovation Center, North Carolina State University, Raleigh, NC, United States

**Keywords:** metabolomic profile, microbiome, plant-growth-promoting microbes, plant-microbiome interaction, stress adaptation

## Abstract

**Introduction:**

Plant–microbiome interactions in the rhizosphere are critical for plant adaptation to environmental stress; however, the coordinated roles of root exudates and microbiome dynamics remain poorly understood.

**Methods:**

Integrating untargeted metabolomics and shotgun metagenomics, we analyzed drought responses in drought-tolerant and drought-sensitive bermudagrass genotypes.

**Results:**

Drought stress shaped the root exudate chemistry, which likely reprogrammed microbiome functions, such as TccC toxins and the Type VI secretion system, without considerable broad taxonomic shifts. A few metabolites, including riboflavin and 1-carboxy-6-hydroxy-3,4-dihydro-beta-carboline, were associated with *Massilia putida*, particularly in the rhizosphere of the drought-tolerant genotype.

**Discussion:**

Our data suggest a potential explanation for a genotype-driven strategy of microbiome modulation via metabolite signaling.

## 1 Introduction

Root metabolites released as root exudates regulate plant–microbiome interactions, particularly under environmental stress. These metabolites, including amino acids, organic acids, sugars, and a range of secondary compounds, are products of plant metabolic activity and serve as precursors to root exudates. A significant portion of plant carbon investment belowground, e.g., up to approximately 25%, can be released into the surrounding soil through exudation (Lamichhane et al., 2024). Root exudation responds to both biotic and abiotic stimuli and creates a chemical-rich niche for the microbial community. Specific exudates function as chemical signals to attract beneficial organisms such as mycorrhizal fungi, N-fixing rhizobia, and plant growth-promoting rhizobacteria (PGPR), while others are simply used as carbon, energy, and nutrient sources for microbial survival and activity. Through these modes of action, root exudates enhance plant resilience and facilitate adaptation to environmental stressors (Faure et al., 2009; Berendsen et al., 2012; Williams and de Vries, 2020).

Drought stress triggers plant adaptive metabolism and leads to considerable metabolic reprogramming by altering carbon allocation, osmolyte production, hormone signaling, and antioxidant defenses (Spikett et al., 1992; Hare et al., 1998; Dennison et al., 2001; Chen and Murata, 2002). In addition, plants modify root exudation chemistry to shape the rhizosphere microbiome. Since root exudates represent the primary chemical interface between plant roots and soil microbes, they play a central role in coordinating plant–microbiome interactions under stress. Drought-induced shifts in exudate composition likely reshape the microbiome and its function via metabolite-mediated selection, influencing the stress resilience of plant genotypes (Canarini et al., 2016; Sasse et al., 2018; Rolfe et al., 2019).

Understanding exudate–microbiome interactions is therefore essential to elucidate how plants actively regulate their microbiomes during water deficit. However, it remains challenging both technically and experimentally. Root exudate collection is complicated by potential microbial contamination, artificial growth conditions, and root damage, which can distort exudate profiles. Furthermore, many studies overlook the functional consequences of microbial shifts under drought. Reductionist experimental designs are often insufficient for disentangling exudate–microbiome interactions in a natural environment that harbors thousands of individual species and metabolites.

To address these gaps, we combined untargeted metabolomics with shotgun metagenomics to examine how genotype-specific metabolic responses to drought influence rhizosphere microbial communities. Using two bermudagrass genotypes, drought-tolerant “TifTuf” and drought-sensitive “Tifway”, we applied an optimized exudate collection strategy across three water regimes and decoded potential interactions between microbial species and root exudates via analyses of two-mode bipartite networks and one-mode bipartite network projections. This integrative approach enables us to determine how drought-induced metabolite shifts shape microbial composition and function and to identify key metabolite–microbe interactions that contribute to drought resilience. By linking plant genotype, metabolic output, and microbial dynamics, our study provides insights into rhizosphere-mediated stress adaptation in plants.

## 2 Materials and methods

### 2.1 Bermudagrass genotypes and experimental setup

Two genotypes of bermudagrass (*Cynodon dactylon*) were used to investigate the influence of plant metabolites on the rhizosphere microbiome under drought. TifTuf is known for its remarkable ability to maintain turf quality under drought, whereas Tifway is less water efficient and thus a drought-susceptible genotype (Schwartz et al., 2018). Sods (i.e., mats of grass on the surface layer of ground) of both genotypes were donated by BuySod (Pinehurst, NC, United States). Uniform sod plugs (15 cm in diameter) were then cut and planted into plastic pots (~ 15 cm deep × ~16 cm in diameter) containing a mixture of sand and professional growing medium (75% Canadian sphagnum peat moss, perlite, dolomitic lime, limestone, and wetting agent) at a 1:3 volume ratio. Grass nutrients were provided weekly using a liquid application of Scotts Turf Builder (The Scotts Miracle-Gro, Marysville, OH, United States) according to the manufacturer’s guidelines. All pots were kept in a greenhouse and watered every other day to promote grass establishment during the first 3 weeks. The grasses were then mowed uniformly to approximately 2.5 cm, and drought stress was initiated by witholding watering, while pots in the control group continued to receive water. Tifway grass began to turn yellow 10 days after witholding watering, signifying that soil water supply fell below evapotranspiration and thus indicating drought stress (Gouveia et al., 2025). Therefore, grass samples collected 10 and 16 days after witholding watering were considered to represent the moderate drought treatment and the severe drought treatment, respectively, to capture drought responses of the rhizosphere microbiome. There was a total of 18 pots, representing two genotypes (TifTuf and Tifway), three watering treatments (control, moderate drought, and severe drought), and three biological replicates per treatment. From each pot, root exudates and root metabolite extracts were collected for downstream metabolomic profiling, while rhizosphere soil samples were harvested for microbiome analysis.

### 2.2 Collection of root exudates and root metabolite extracts

Root exudates were collected using a modified sterile hydroponic protocol to minimize biotic and abiotic interference from soil (Lucas García et al., 2001; Canarini et al., 2019; Otxandorena-Ieregi et al., 2024). Briefly, entire bermudagrasses were gently uprooted and thoroughly rinsed with tap water to remove adhering sand and growth medium. Only undamaged roots were retained for collecting exudates, since intracellular compounds might leak from wounded roots. Roots were surface sterilized by sequential immersion in 70% ethanol for 1 min, followed by a Micropur Classic solution (5 mg/L; Katadyn®, Switzerland) for 1 min to reduce microbial presence. To minimize osmotic shock and stabilize physiological conditions, roots were preconditioned in 50% Hoagland nutrient solution for 15 min before final transfer to sterile Milli-Q water for exudate collection.

Root systems were submerged in 100 mL of sterile Milli-Q water to collect exudates for 3 h at room temperature (~23 °C) under dark conditions, as longer period might result in the accumulation of exudates under far-original metabolic conditions, such as artificially altering root cell viability, reducing the likelihood of bacterial growth, and limiting the reabsorption of root exudates (Reilly et al., 2003; Jones et al., 2004; Döll et al., 2024). Following 0.2 μm filtration, filtrates were stored at −20 °C prior to the metabolomic analysis. Root metabolic extracts were collected from approximately 100 mg of frozen root tissue after being ground into fine powder in liquid nitrogen. Extraction was performed sequentially by adding 2 mL of chilled 50% (v/v) methanol, followed by homogenization, incubation at 65 °C for 40 min, brief vortexing, and centrifugation at 16000×g for 5 min. The resulting supernatant was filtered to 0.2 μm and stored at −20 °C prior to metabolomic analysis.

### 2.3 High-performance liquid chromatography–mass spectrometry (HPLC-MS) for metabolomic analysis

Both root exudates and root metabolite extracts were quantified via high-performance liquid chromatography coupled with mass spectrometry (HPLC-MS) at the Molecular Education, Technology and Research Innovation Center (METRIC) of North Carolina State University. The samples were first lyophilized and then reconstituted in a 1:1 water:methanol solution containing Waters QC Reference. Quality assurance was maintained using solvent blanks, neat QC standards, mock extraction blanks, and pooled QC injections.

HPLC-MS analysis was performed using a Thermo Fisher Scientific Vanquish UHPLC system (Germering, Germany), as described previously (Krakko et al., 2026). Reversed-phase LC was performed on a Waters Acquity Premier BEH C18 column (100 × 2.1 mm, 1.7 μm particle size). Mobile phase A was 0.1% formic acid in 95:5 water:acetonitrile, and mobile phase B was 0.1% formic acid in acetonitrile. The chromatographic gradient was as follows: 0 min (0% B), 1 min (0% B), 18.0 min (100% B), 22.0 min (100% B), 22.1 min (0% B), 25.0 min (0% B). The column temperature was maintained at 40 °C, with a flow rate of 0.3 mL·min^−1^ and an injection volume of 2 μL. Mass spectrometric detection was performed on a Thermo Fisher Scientific Orbitrap ID-X Tribrid mass spectrometer using both positive and negative ionization modes in separate runs. The scan range was *m*/*z* 70–700. Data-dependent MS^2^ scans were acquired using both HCD (Higher-energy Collisional Dissociation) and CID (Collision-Induced Dissociation) fragmentation. Internal mass calibration was achieved using Thermo Scientific EASY-IC Ion source system.

### 2.4 Rhizosphere soil collection and DNA extraction for shotgun sequencing

Rhizosphere samples were prepared for both TifTuf and Tifway following a modified bleach-washing protocol (Xia et al., 2021). Metagenomic DNA of each soil sample (~250 mg) was extracted using the DNeasy PowerSoil Pro Kit (Qiagen, Venlo, Netherlands) according to the manufacturer’s recommended protocols. DNAs extracted from the three replicates of each treatment were normalized to the same concentration and pooled in equal volumes into a composite DNA sample for shotgun paired-end sequencing in the Genomic Sciences Laboratory (GSL) of North Carolina State University using the Illumina NovaSeq 6,000 platform. All sequencing data were deposited in the NCBI BioProject database under accession number PRJNA1403647.

### 2.5 Metabolomics and metagenomics annotation

HPLC-MS data annotation was performed using Compound Discoverer 3.4 (Thermo Fisher Scientific), with putative compound identifications based on accurate mass, isotopic pattern, and MS/MS fragmentation against various spectral libraries and databases (including an in-house spectral library using mzVault, mzCloud, ChemSpider, and MassList nodes). Annotation confidence was assessed using a custom workflow node within Compound Discoverer, as described previously (Krakko et al., 2026). Compounds with an annotation confidence of Level 4 or 5 were filtered out. A custom R script was used to merge the positive and negative dataset exports from Compound Discoverer. Annotated compounds were classified by classyfireR (v0.3.8) using the compound-unique InChiKey. Multiple peaks in the untargeted metabolomics exports may be assigned the same compound name with different mass-to-charge ratios due to different adducts, isotopes, structural isomers, or the inherent ambiguity of lower-confidence identifications (i.e., Levels 2 and 3). These entries remained separate for downstream statistical and multivariate analyses to keep critical biochemical information (Worley and Powers, 2013).

A bioinformatic pipeline was used to annotate paired-end sequencing reads. Raw reads (65231336–91063724) were subjected to KneadData (v0.12.1) analysis to remove possible plant DNAs with the assembled genome of bermudagrass as the reference (Wang et al., 2024) and to perform quality checks. On average, 0.2% of raw reads were of plant origin, and ~12% of raw reads failed quality filtering and trimming. Therefore, ~88% of high-quality reads were used for taxonomic classification using Kaiju (v1.9.0) against the pre-built indices, including RefSeq (August 4, 2024) for bacteria and Fungi (16 August 2024) for fungi.

The high-quality reads were further assembled into contigs using MEGAHIT (Li et al., 2015). The obtained contigs were used for predicting protein-coding genes by MetaGeneMark-2. A non-redundant gene catalog was then constructed using CD-HIT (Li and Godzik, 2006). Alignment of sample DNA sequences with unigenes was performed using Bowtie (Langmead, 2009), and aligned sequences were sorted and indexed using SAMtools (Danecek et al., 2001). The relative abundance of each gene in a sample was calculated as the copy number divided by the sum of copy numbers of all genes mapped in this sample. Finally, genes were annotated for functions based on the KEGG orthology system by searching the KOfam database (using hidden Markov models [HMMs]) using PykofamSearch.

### 2.6 Statistical analysis

Principal coordinates analysis (PCoA) was used on metabolomic and metagenomic datasets to determine if data points were similar enough to cluster by bermudagrass genotype and drought stress using the Bray–Curtis dissimilarity. Statistical significance was validated via adonis, a robust permutational multivariate analysis of variance. Because DNA samples of three replicates were pooled together as a composite DNA sample due to financial constraints, we opted to use a randomized block design to compare genotype effects with drought stress severities as blocks and vice versa for comparing drought effects. The use of composite samples for shotgun sequencing reduced statistical power, limiting our ability to detect significant main effects of genotype and drought on taxon and functional gene abundances. Thus, we reported *p*-values between 0.05 and 0.10 to demonstrate the occurrence of main effects that may have occurred by random chance. In addition, interactions between genotype and drought could not be examined for metagenomic datasets but could be for metabolomic datasets since replicates were analyzed separately. The Mantel test was further used to assess the correlation between Bray–Curtis distance matrices to infer how plant metabolites were associated with the rhizosphere microbiome and how microbial taxa affected the community function.

A bipartite network was constructed to detail correlations between metabolites and specific microbial species and functions, and significant connections were extracted by backbone projections. Differentially represented metabolites and functional genes were identified using edgeR. The previously described block design was used, indicating that drought treatments were compared with genotypes as blocks (e.g., design = model.matrix(~genotypes + drought). Likelihood ratio tests (LRT) were used for comparison based on a false discovery rate (FDR) of <0.05. Because edgeR is designed to model data using a negative binomial distribution, raw read count data were used and filtered to ensure statistical power per genotype. All statistical analyses were carried out using packages in R, including vegan (v2.7-1), edgeR (v4.5.1), bipartite (v.2.21), and backbone (v2.1.4). Statistical significance was reported at a *p*-value of <0.05 or an FDR-value of <0.05, unless indicated otherwise.

## 3 Results

**Figure 1:**
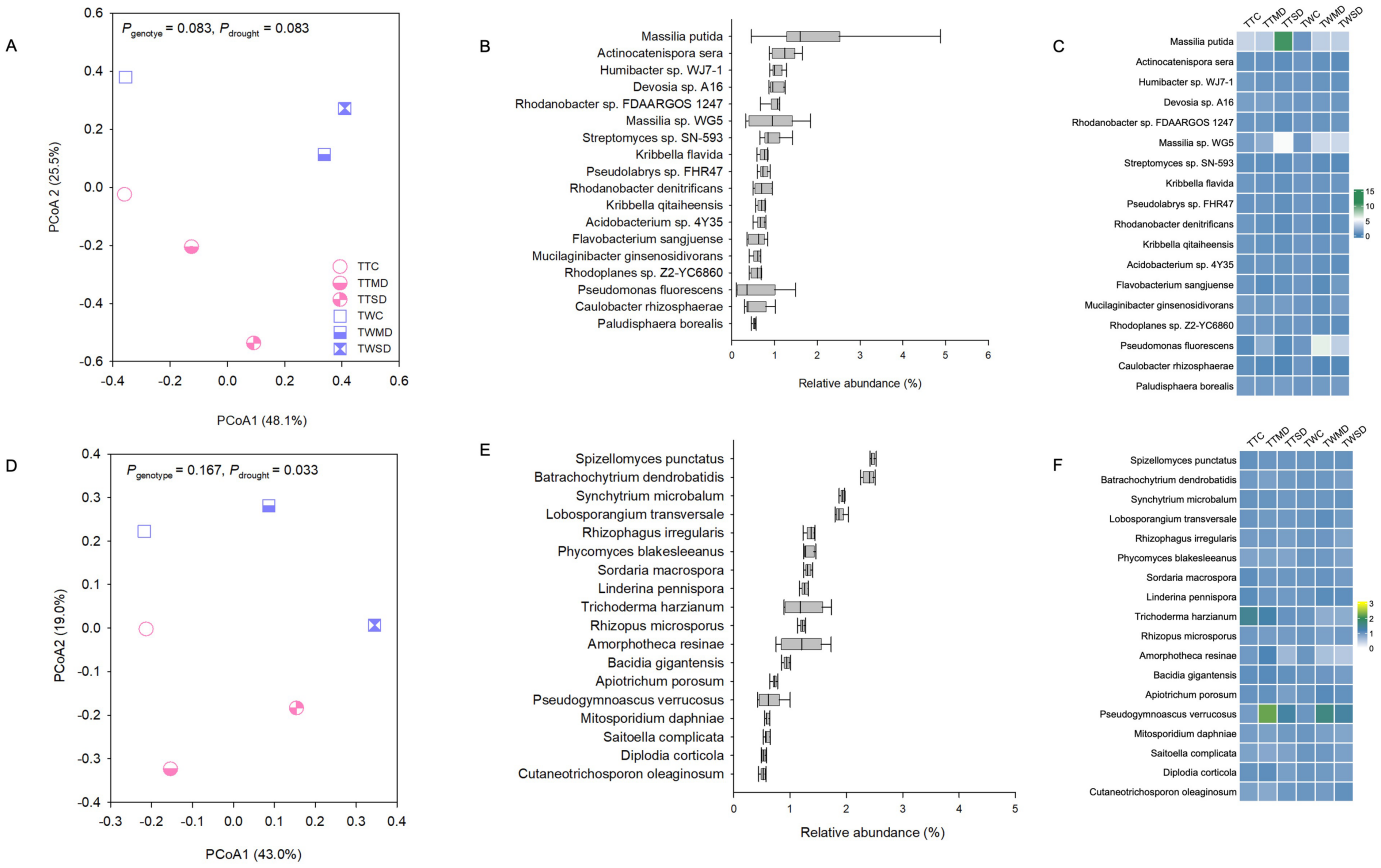
Bacterial and fungal species compositions. **(A,D)** Principal coordinates analysis to visualize species compositional differences for bacteria and fungi, respectively, between two Bermudagrass genotypes, TifTuf (TT) and Tifway (TW) and among three watering conditions, control (C), moderate drought (MD), and severe drought (SD). **(B,E)** The mean values of the relative abundance of bacterial and fungal species having >0.5% relative abundance, respectively, across two genotypes and three soil watering levels. **(C,F)** Heatmaps for bacteria and fungi, respectively, comparing differences in the relative abundance of abundant species (i.e., >0.5%) among two genotypes and three watering moisture levels.

Genotype and drought tended to shape both rhizosphere bacterial and fungal communities at *p* = 0.083 (Figures 1A,D). Samples were grouped by drought along PCoA1 (48.1% of the variance), suggesting a drought-driven shift in both bacterial and fungal community compositions. On average, Proteobacteria was the dominant phylum (~55%), followed by Actinobacteria (~30%) and Bacteroidetes (~10%) (Supplementary Figure 1). Alphaproteobacteria (~30%), Betaproteobacteria (~20%), and Gammaproteobacteria (~10%) were the dominant Proteobacterial classes. Some other phyla, including Planctomycetes, Acidobacteria, Firmicutes, Verrucomicrobia, Chloroflexi, and Gemmatimonadetes, were also detected, although each represented less than 5% of the total abundance, collectively contributing to community heterogeneity. *Massilia* of Betaproteobacteria was the most abundant genus (Figure 1B). Drought appeared to enrich *Massilia putida* and *Massilia* sp., with this effect being more obvious in the rhizosphere of drought-tolerant bermudagrass TifTuf under severe drought (Figure 1C). Additionally, drought tended to promote *Pseudomonas fluorescens* in the rhizosphere of drought-sensitive bermudagrass Tifway. The fungal community was dominated by Ascomycota (~60%), which could be attributed to enrichment of Sordariomycetes and Eurotiomycetes, followed by Basidiomycota (~20%), represented primarily by Agaricomycetes and Tremellomycetes (Supplementary Figure 2). *Pseudogymnoascus verrucosus* accounted for ~1.2% and exhibited a consistent yet distinct pattern of relative abundance across drought treatments in the two genotypes, being most abundant under moderate drought and lowest in well-watered conditions (Figures 1E,F).

**Figure 2:**
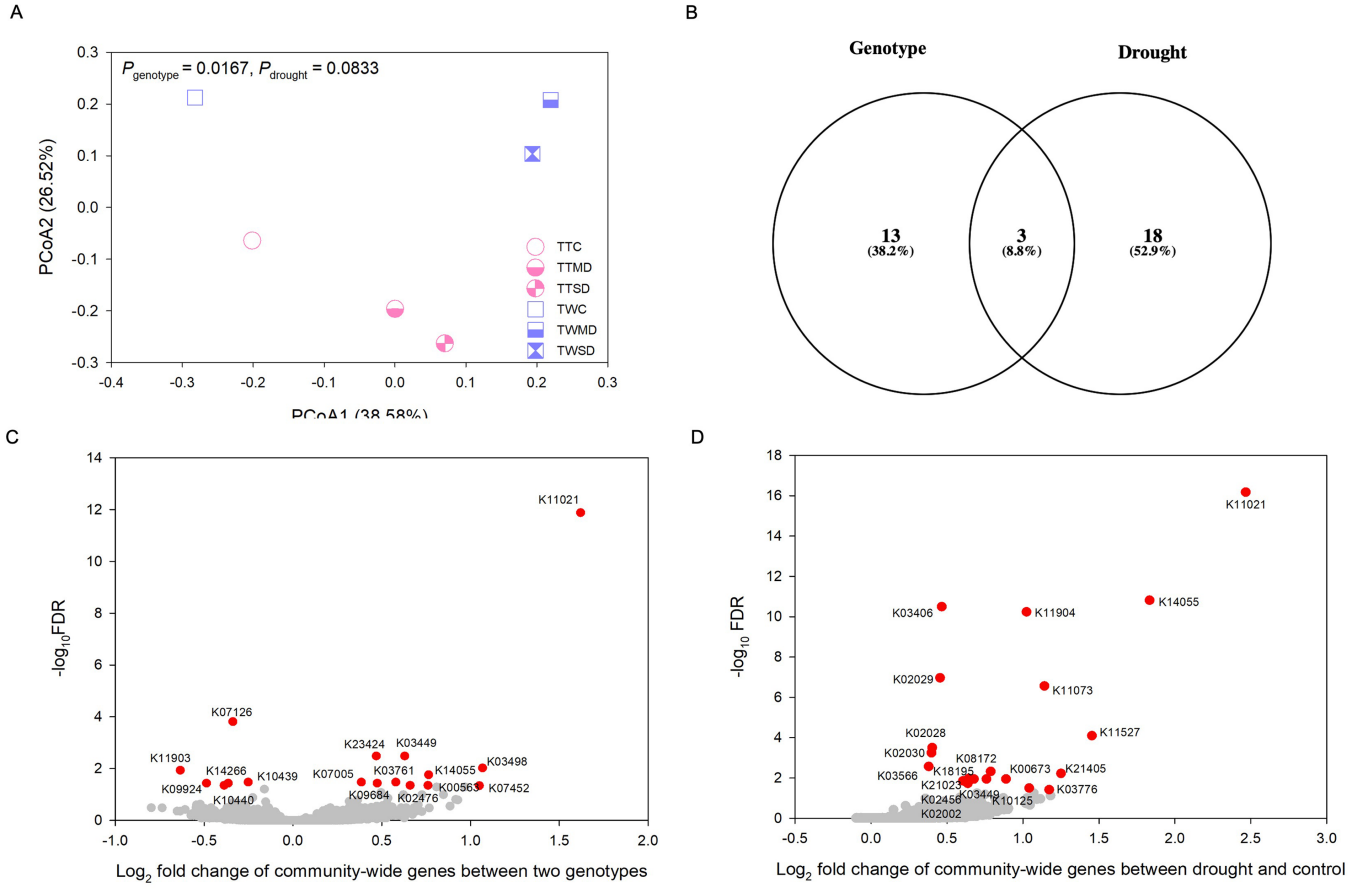
Microbial functional genes. **(A)** Community-wide differences in KO numbers between TifTuf (TT) and Tifway (TW) genotypes and between control (C), moderate drought (MD), and severe drought (SD). **(B)** A Venn diagram showing the number and percentage of functional genes that differed between genotypes and between drought treatments. **(C,D)** Volcano plots showing logarithmic fold changes in counts per million (CPM) between the two genotypes and between control and the average of MD and SD, respectively, at a false discovery rate (FDR) of <0.05.

Microbial gene abundance also showed significant differences by genotype (*p* = 0.0167) and marginal differences by drought (*p* = 0.0833) (Figure 2A). A total of 16 Kyoto Encyclopedia of Genes and Genomes (KEGG) orthology identifiers (i.e., K numbers) varied significantly between genotypes, and 21 responded to drought stress (FDR < 0.05, Figure 2B). Among these, K11021, encoding the insecticidal toxin complex protein TccC, differed most strongly for both genotype and drought comparisons (Figures 2C,D). Other notably differentiated genes included K14055 (universal stress protein E), K11904 (type VI secretion system secreted protein VgrG), and K11073 (putrescine transport system substrate-binding protein). Three functional genes, K11021, K14055, and K03449 (encoding major facilitator superfamily transporter, cyanate permease family, and cyanate transporter), were responsive to both genotype and drought (Figure 2B). In contrast, 13 functional genes were uniquely responsive to genotypes, and 18 were specific to drought treatments.

**Figure 3:**
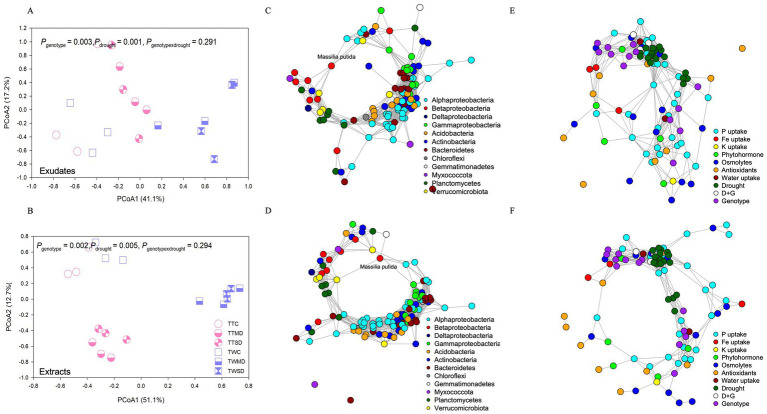
**(A,B)** Principal coordinates analysis showing the separation and clustering of root extract and root exudate samples between TifTuf (TT) and Tifway (TW) bermudagrass genotypes and among control (C), moderate drought (MD), and severe drought (SD). **(C,D)** Bipartite network projections extracted from positive and negative connections between bacterial species and root exudate compounds, respectively. **(E,F)** Bipartite network projections extracted from positive and negative connections between KEGG orthology identifiers (i.e., K numbers) and root exudate compounds, respectively.

Both root extract and root exudate metabolomic profiles exhibited distinct shifts associated with bermudagrass genotypes (*p* = 0.003) and drought treatment (*p* = 0.001) (Figures 3A,B). The separation between control and drought was more pronounced in extracts than in exudates. Significant correlations were found among the root metabolite profile, bacterial community composition, and the rhizosphere metagenomic function (Supplementary Figure 3), with complex relationships characterized and visualized by bipartite networks (Supplementary Figures 4 and 5). The overall connection (i.e., the sum of positive and negative connectance) between root exudates and bacterial species or between root exudates and functional genes was relatively low, accounting for ~11% of the theoretically possible connections (Supplementary Table 1). Bipartite network projections were constructed based on both positive and negative bipartite networks to characterize the similarity or dissimilarity of bacterial species’ responses to root exudates (Figures 3C,D). Species of Alphaproteobacteria, Acidobacteria, and Bacteroidetes formed a densely connected group, while species of Betaproteobacteria, Planctomycetes, and Verrucomicrobia were represented by a different cluster, suggesting differences in their response to root exudates. Bipartite network projections were also performed to compare the similarity or dissimilarity in gene responses to root exudates (Figures 3E,F). Genes included for analysis were those showing significant differences between genotypes and drought treatments and those involved in phosphorus solubilization, iron and potassium transport, antioxidant defense, osmolarity, and phytohormone biosynthesis that presumably helped plant adaptation to drought. Approximately one-third of genes for phosphorus solubilization/uptake or osmolyte production were clustered with genotype- and drought-responsive genes in response to root exudates. Two genes for water uptake were also clustered with genotype- and drought-responsive genes. By contrast, <10% of genes for antioxidant defense, phytohormone homeostasis, and siderophore and K uptake were grouped with genotype- and drought-responsive genes.

Root exudates that promoted Alphaproteobacterial species with >0.1% relative abundance were different from those associated with Betaproteobacterial species (Supplementary Table 2). Only eight root exudate compounds showed significant and positive correlations with approximately half of these Alphaproteobacterial species. By contrast, >60 root exudate compounds were positively associated with approximately half of Betaproteobacterial species. Genotype- and drought-responsive genes also showed differential responses to root exudates (Supplementary Table 3), with genotype-responsive genes reacting to most root exudates of the class fatty acyls, while drought-responsive genes reacted to compounds of more diverse classes, such as carboxylic acids, hydroxylic acids, fatty acyls, and naphthalenes. Three genes that were both genotype- and drought-responsive were positively correlated with 85 root exudate compounds, including indoles and derivatives (Supplementary Table 4). *M. putida* of Betaproteobacteria that was apparently enriched in drought-tolerant bermudagrass, TifTuf, was positively associated with only 10 root exudate compounds (Table 1). Riboflavin was positively associated with *M. putida* and a few other species, but the compound 1-carboxy-6-hydroxy-3,4-dihydroxy-beta-carboline of the class alkaloids was solely associated with *M. putida*.

**Table 1:** Root exudates showing significant positive correlations with *Massilia putida*, the most enriched species in the rhizosphere of TifTuf under severe drought.

Exudates	Superclass	Class	Other species with positive relationships
Colletotrialide^3b^	Organoheterocyclic compounds	Benzofurans	*Bosea* sp. *AS-1* (Alphaproteobacteria)
			*Caulobacter segnis* (Alphaproteobacteria)
			*Dyella terrae* (Gammaproteobacteria)
			*Flavisolibacter ginsenosidimutans* (Bacteroidetes)
4-methylhippuric acid^3b^	Benzenoids	Benzene and substituted derivatives	*Phenylobacterium zucineum* (Alphaproteobacteria)
			*Mesorhizobium* sp. *B2-8-5* (Alphaproteobacteria)
1-carboxy-6 hydroxy-3,4-dihydro-beta-carboline^3b^	Alkaloids and derivatives	Harmala alkaloids	
Methyl 4-amino-2-(2,3-dihydroxy-3-methylbutyl)benzoate^3b^	—	—	*Phenylobacterium zucineum* (Alphaproteobacteria)
			*Mesorhizobium* sp. *B2-8-5* (Alphaproteobacteria)
4-((1-acetoxy-3-methylpentan-2-yl)amino)-3-methyl-4-oxobutanoic acid^3b^	Organoheterocyclic compounds	Isobenzofurans	*Bosea* sp. *AS-1* (Alphaproteobacteria)
			*Caulobacter segnis* (Alphaproteobacteria)
			*Dyella terrae* (Gammaproteobacteria)
			*Flavisolibacter ginsenosidimutans* (Bacteroidetes)
O-suberoylcarnitine^3b^	Organic acids and derivatives	Carboxylic acids and derivatives	*Bosea* sp. *AS-1* (Alphaproteobacteria)
			*Caulobacter segnis* (Alphaproteobacteria)
			*Dyella terrae* (Gammaproteobacteria)
			*Flavisolibacter ginsenosidimutans* (Bacteroidetes)
Amicoumacin A^3b^	Organic acids and derivatives	Carboxylic acids and derivatives	*Streptomyces* sp. *SN-593* (Actinobacteria)
			*Phenylobacterium zucineum* (Alphaproteobacteria)
			*Mesorhizobium* sp. *B2-8-5* (Alphaproteobacteria)
Protostemonine^3b^	Alkaloids and derivatives	Stemona alkaloids	*Streptomyces* sp. *SN-593* (Actinobacteria)
			*Streptomyces* sp. *So13.3* (Actinobacteria)
			*Sorangium cellulosum* (Myxococcota)
4-Methylene-2-octyl-5-oxotetrahydrofuran-3-carboxylic acid^3b^	Organoheterocyclic compounds	Lactones	*Phenylobacterium zucineum* (Alphaproteobacteria)
			*Caulobacter segnis* (Alphaproteobacteria)
			*Mesorhizobium* sp. *B2-8-5* (Alphaproteobacteria)
Riboflavin^2^	Organoheterocyclic compounds	Pteridines and derivatives	*Phenylobacterium zucineum* (Alpharoteobacteria)
			*Bosea* sp. *AS-1* (Alphaproteobacteria)
			*Mesorhizobium* sp. *B2-8-5* (Alpharoteobacteria)

Some of these root exudates were also positively correlated with species belonging to Alphaproteobacteria, Bacteroidetes, Actinobacteria, and other phyla. The superscript following an exudate compound indicates the confidence level of identification. Level 3b: Matched to a compound database; usually, several putative compound names can be associated with the compound, but the reported name represents the highest match; Level 2: Matched to a spectral library. The symbol “—” indicates no classification in the R package classyfireR.

## 4 Discussion

Our results demonstrate that the host genotype is a key determinant of rhizosphere microbiome structure, particularly under stress. *M. putida* was likely enriched by severe drought in the rhizosphere of the drought-tolerant genotype TifTuf. The ability of *Massilia* to tolerate stress has been documented in various extreme and oligotrophic environments, such as deserts and rock surfaces (Ofek et al., 2012; Ren et al., 2018; Manni and Filali-Maltouf, 2022), highlighting its ecological resilience. Recognized as a plant-beneficial taxon, *Massilia* is associated with traits such as stimulation of lateral root formation, enhancement of nitrogen acquisition, and overall stress tolerance (Yu et al., 2021; He et al., 2024; Li et al., 2025). Therefore, the apparent enrichment of *M. putida* in TifTuf under drought suggests that this genotype likely recruits beneficial microbes as a core component of its drought tolerance strategy.

Such selection for *M. putida* is likely driven by chemical communication in the rhizosphere. A metabolomic analysis revealed highly specific and selective compound–microbe associations. Of the 1,768 peaks detected in the root exudates, only ~11% exhibited statistically significant correlations with abundant bacterial species, suggesting chemical filtering of microbial taxa. Notably, Alphaproteobacterial and Betaproteobacterial species showed distinct responses to the rhizosphere chemical environment, echoing their contrasting life history strategies. The abundant species of Alphaproteobacteria, often acting as oligotrophs, were correlated with a narrow subset of rhizosphere chemicals, such as water-insoluble compounds. By contrast, the abundant species of Betaproteobacteria, generally deemed to be copiotrophs, were significantly correlated with numerous water-soluble metabolites. These findings suggest that specific microbial groups can be selectively modulated via targeted exudate signals without broadly disrupting the surrounding microbiome. Therefore, we hypothesized that microbe–metabolite interactions provide a potential strategy to modulate microbial function in the rhizosphere through minimal chemical inputs, enabling fine-tuned manipulation of plant–microbiome systems under stress.

Ten compounds in the root exudates were identified as being positively correlated with the relative abundance of *M. putida,* one of which, 1-carboxy-6-hydroxy-3,4-dihydroxy-beta-carboline, was uniquely associated with *M. putida* and no other abundant species. Whether this association is due to this species’ metabolic capability to catabolize this compound or to a more active plant-mediated signaling pathway remains unclear but is supported by prior studies showing *Massilia*’s role in enhancing drought tolerance via ROS scavenging, aquaporin upregulation, and osmolyte production (He et al., 2024; Li et al., 2025). Beta-carbolines and their derivatives are often increasingly produced by plants under drought as an adaptive mechanism (Guo et al., 2025). Riboflavin, a metabolite known for improving plant drought tolerance by reducing oxidative stress (Deng et al., 2014), was also correlated with *M. putida* and a few other microbial species. In this study, the strong associations between apparently independent observations, i.e., the abundant presence of *M. putida* in the rhizosphere and the detection of specific plant metabolites known to be enhanced under drought, enhance the possibility of a specialized signaling mechanism, in which host-derived secondary metabolites likely facilitate the recruitment of stress-resilient microbial taxa.

*Streptomyces* is often reported to dominate under drought (De Vries et al., 2020). In our previous field study, the phylum Actinobacteria and its genus *Streptomyces* were enriched in the rhizosphere and root endosphere of bermudagrass under water deficit and were putatively associated with reduced ethylene levels and reactive oxygen species (ROS) scavenging (Hu et al., 2023). Despite differences in technique and resolution (shotgun sequencing versus amplicon sequencing), Actinobacteria and *Streptomyces* were abundant in the rhizosphere of both genotypes. However, most *Streptomyces* spp. were not differentially enriched by drought between the two genotypes, suggesting that plants employ a blended strategy to target microbes that confer drought tolerance regardless of genotype and those that enhance genotype-specific drought resilience.

In parallel with possible genotype-driven differences in the assemblage of the microbial community, several microbial functional genes were significantly influenced by genotype and drought, including the toxin complex protein TccC (K11021). While originally characterized as an insect toxin, TccC has since been detected in the plant-associated microbiome, showing no consistent correlation with insecticidal activity but possible involvements in competition for resources and defense against pathogens (Waterfield et al., 2001; Dowling and Waterfield, 2007; Rangel et al., 2016; Wemheuer et al., 2017). In our study, the enrichment of TccC abundance suggests roles in modulating the rhizosphere environment, mediating stress response, and thus likely helping enhance plant fitness. This deduction was in line with reports of TccC presence in other drought-adapted rhizospheres (Sarkar et al., 2022).

Similarly, the VgrG protein (K11904), a key component of the Type VI secretion system (T6SS) that enhances microbial competence in the rhizosphere by primarily shaping species competition (Bernal et al., 2018; Coulthurst, 2019; Boak et al., 2022), has also been implicated in enhancing plant–microbiome interactions and plant environmental fitness. For instance, root exudates activated T6SS of *Rhizobium etli* to promote its root colonization, nodule development, and therefore plant performance (Salinero-Lanzarote et al., 2019). Nonetheless, the simultaneous enhancement of K11021, K11904, and K14055, a universal stress protein by drought stress, suggests that they contribute to microbial fitness and persistence under stressful conditions. Drought seems to intensify microbial competition and prompt microbes to engage in defense or colonization strategies to persist in the rhizosphere (Durán et al., 2021; Allsopp and Bernal, 2023; Garin et al., 2025). Whether these systems primarily act as competitive tools or facilitate beneficial interactions with the plant remains to be clarified.

By comparing cellular and tissue chemicals for plants with or without microbial inoculants, empirical data have led to inferences about why microbes can mitigate plant drought stress. The inference that has received the most research attention is the microbial regulation of phytohormones, nutrient acquisition, and scavenging of reactive oxygen species through the synthesis of antioxidant enzymes. However, these genetic responses to root exudates varied considerably, with some responding similarly to drought- or genotype-responsive genes, including genes for P solubilization and uptake, water transport and uptake, and osmolyte regulation, as well as antioxidant activity. These, together with findings on K11021, K11904, and K14055, suggest that multiple microbial functions or mechanisms contribute to plant drought resilience.

In summary, our study demonstrates that bermudagrass initiates stress adaptation through shifts in its metabolite profile. These changes elicit beneficial microbes to further promote plant plasticity to stress. While both genotypes almost indifferently boost Actinobacteria, specifically its genus *Streptomyces*, a common drought-adaptive microbial taxon, the drought-tolerant genotype appears to have an edge over the drought-sensitive genotype in recruiting additional drought-adaptive and plant growth-promoting microbes. A few metabolites, such as 1-carboxy-6-hydroxy-3,4-dihydro-beta-carboline and riboflavin, were identified to target the selection of *M. putida* and therefore microbial functions, suggesting a possible genotype-driven strategy of microbiome modulation via metabolite signaling.

## Data Availability

The datasets presented in this study can be found in online repositories. The names of the repository/repositories and accession number(s) can be found in the article/Supplementary material.
