# Supplementary material for: Rhizosphere microbiomes in drought-tolerant and drought-sensitive bermudagrass genotypes: root exudate association
Source: Front Microbiol. 2026 Jul 23;17:1868900. doi: 10.3389/fmicb.2026.1868900 (PMC13442629; doi:10.3389/fmicb.2026.1868900)
Supplement: Supplementary file 1 [file Table_1.DOCX]

**Rhizosphere microbiomes in drought-tolerant and drought-sensitive bermudagrass genotypes: root exudate selection**

Sayada Momotaz Akther^1^, Daniel Krakko^2^, and Wei Shi^1^

^1^Department of Crop and Soil Sciences, North Carolina State University, Raleigh, NC 27695

^2^The Molecular Education, Technology and Research Innovation Center, North Carolina State University, Raleigh, NC 27695

Supplementary Table: 4

Supplementary Figure: 5

**Supplementary Table 1.** Parameters of bipartite networks between the metabolomic profile of root exudates and bacterial species (or KEGG orthology identifiers, i.e., K numbers). A total of 117 bacterial species with > 0.1% relative abundance were included in the analysis. Ninety-five K numbers for the analysis were those showing significant differences (FDR < 0.05) between genotypes and between drought treatments and those involved in P, K, Fe, and water uptake, osmolyte biosynthesis, antioxidant activity, and phytohormone regulation.

|  | Species-exudate bipartite | | KEGG-exudate bipartite | |
| --- | --- | --- | --- | --- |
|  | Positive | Negative | Positive | Negative |
| Connectance | 0.052 | 0.073 | 0.061 | 0.040 |
| Nestedness | 4.765 | 11.495 | 9.214 | 3.576 |
| Robustness.HL | 0.973 | 0.978 | 0.973 | 0.968 |
| Robustness.LL | 0.758 | 0.813 | 0.763 | 0.706 |
| Links per species | 5.625 | 7.931 | 5.473 | 3.625 |

**Supplementary Table 2.** Some root exudates correlated with >40% Alphaproteobacterial species or >40% Betaproteobacterial species. Of 1,768 root exudate compounds (peaks) detected via HPLC-MS, 530 and 600 were positively correlated (i.e., Spearman rank correlation coefficient ≥ 0.7 and *P* < 0.05) with a species belonging to Alphaproteobacteria and Betaproteobacteria, respectively. However, only eight and 63 exudate compounds were correlated with > 40% of abundant species (> 0.1% relative abundance) of Alphaproteobacteria and Betaproteobacteria, respectively. The superscript following an exudate compound indicates the confidence level of identification; level 3b: matched to a compound database; usually there are several putative compound names that can be associated with but the reported name represents the highest match; level 3a: having more than one Level 1 or Level 2 matches; level 2: matched to a spectral library.

|  | Superclass | Class |
| --- | --- | --- |
| Root exudates correlated with Alphaproteobacterial species | | |
| (7S,10aS)-7-Pentyloctahydro-2H-pyrido[1,2-d][1,4]oxazepin-2-one^3b^ | Organoheterocyclic compounds | Oxazepines |
| 9S,13R-12-Oxophytodienoic acid^3a^ | Lipids & lipid-like molecules | Fatty acyls |
| Palmitoyl ethanolamide^3b^ | Organic acids & derivatives | Carboximidic acids & derivatives |
| 3-Hydroxytridecanoyl carnitine^3b^ | Lipids & lipid-like molecules | Fatty Acyls |
| Piptamine(antibiotic)^3b^ | Alkaloids and derivatives | Lupin alkaloids |
| Pipereicosalidine^3b^ | Organoheterocyclic compounds | Piperidines |
| 16,17-Dihydroxykauran-18-oic acid^3a^ | Lipids & lipid-like molecules | Prenol lipids |
| Caffeic acid^3a^ | Phenylpropanoids & polyketides | Cinnamic acids & derivatives |
| Some root exudates correlated with Betaproteobacterial species | | |
| Valylproline^2^ | Organic acids & derivatives | Carboxylic acids & derivatives |
| L-Asparaginyl-L-isoleucine^3b^ | Organic acids & derivatives | Carboxylic acids & derivatives |
| Isoprenaline^3b^ | Benzenoids | Phenols |
| Glu-Lys^3b^ | - | - |
| N-Feruloylglycine^3b^ | Organic acids & derivatives | Carboxylic acids & derivatives |
| Tiglylcarnitine^3b^ | Lipids & lipid-like molecules | Fatty Acyls |
| leu-ala-ser^3b^ | Organic acids & derivatives | Carboxylic acids & derivatives |
| Cicloxilic acid^3b^ | Organic oxygen compounds | Organooxygen compounds |
| Thr-phe^3b^ | Organic acids & derivatives | Carboxylic acids & derivatives |
| Leu-gly-pro^3b^ | Organic acids & derivatives | Carboxylic acids & derivatives |
| Leu-ala-asp^3b^ | Organic acids & derivatives | Carboxylic acids and derivatives |
| Catalpol^3b^ | Organic oxygen compounds | Organooxygen compounds |
| Dihydrophaseic acid 4-O-β-D-glucoside^3b^ | Lipids & lipid-like molecules | Prenol lipids |

**Supplementary Table 3.** Grass root exudate compounds correlated with functional genes that differed significantly between the two genotypes or between drought treatments. Of 1,768 root exudate compounds detected via HPLC-MS, 867 and 638 were positively correlated (i.e., Spearman rank correlation coefficient ≥ 0.7 and P < 0.05) with at least one genotype- or drought-responsive gene, respectively. However, only six and eight exudate compounds were correlated with more than >40% and >75% of genotype- or drought-responsive genes, respectively. The superscript following an exudate compound indicates the confidence level of identification; level 3b: matched to a compound database; usually there are several putative compound names that can be associated with but the reported name represents the highest match; level 3a: having more than one Level 1 or Level 2 matches; level 2: matched to a spectral library; level 1: verified using a reference standard. The symbol “-” indicates no classification in the database of the R package, classyfireR.

|  | Superclass | Class |
| --- | --- | --- |
| Root exudates correlated with genotype-responsive genes | | |
| 9(S)-HpOTrE ^3b^ | Lipid and lipid-like molecules | Fatty acyls |
| 9(S)-HPODE^3b^ | Lipid and lipid-like molecules | Fatty acyls |
| 12,13-DiHOME^3b^ | Lipid and lipid-like molecules | Fatty acyls |
| 9,10-dihydroxy-12Z-octadecenoic acid^3a^ | Lipid and lipid-like molecules | Fatty acyls |
| (15Z)-9,12,13-Trihydroxy-15-octadecenoic acid^2^ | Lipid and lipid-like molecules | Fatty acyls |
| N-Acetyl-L-isoleucine^1^ | Organic acids and derivatives | Carboxylic acids and derivatives |
| Root exudates correlated with drought-responsive genes | | |
| 7-Methyl-1,2-dihydro-1,2-naphthalenediol^3b^ | Benzenoids | Naphthalenes |
| Leu-pro^3b^ | Organic acids and derivatives | Carboxylic acids and derivatives |
| 3-Hydroxysebacic acid^3b^ | Organic acids and derivatives | Hydroxy acids and derivatives |
| (2S,3S,4S)-4-Methyl-5-oxo-2-pentyltetrahydro-3-furancarboxylic acid^3b^ | Organoheterocyclic compounds | Lactones |
| Oscr#19^3b^ | Lipid and lipid-like molecules | Fatty acyls |
| 3,3-Dimethylglutaric acid^3a^ | Lipid and lipid-like molecules | Fatty acyls |
| 5-(1-Amino-2-hydroperoxyethyl)-1,3-benzenediol^3b^ | - | - |
| N-Acetyl-L-leucine^3a^ | Organic acids and derivatives | Carboxylic acids and derivatives |

**Supplementary Table 4.** Some of 85 grass root exudate compounds correlated with all three function genes (K11021, K14055, and K03449) that differed significantly between two genotypes and between drought treatments. The superscript following an exudate compound indicates the confidence level of identification; level 3b: matched to a compound database; usually there are several putative compound names that can be associated with but the reported name represents the highest match; level 3a: having more than one Level 1 or Level 2 matches; level 2: matched to a spectral library; level 1: verified using a reference standard.

|  | Superclass | Class |
| --- | --- | --- |
| Valylhydroxyproline^3b^ | Organic acids & derivatives | Carboxylic acids & derivatives |
| 6-Methylquinoline^3a^ | Organoheterocyclic compounds | Quinolines & derivatives |
| Indole-3-lactic acid^3b^ | Organoheterocyclic compounds | Indoles & derivatives |
| D-Tryptophan^1^ | Organoheterocyclic compounds | Indoles & derivatives |
| 3,4-Methylenedioxy PV8^2^ | Benzenoids | Benzene & substituted derivatives |
| 12-Oxo phytodienoic acid^3a^ | Lipids & lipid-like molecules | Fatty Acyls |
| α-Eleostearic acid^3a^ | Lipids & lipid-like molecules | Fatty Acyls |
| Pinolenic acid^3a^ | Lipids & lipid-like molecules | Fatty Acyls |
| 9-Oxo-ODE^3a^ | Lipids & lipid-like molecules | Fatty Acyls |
| 4-Indolecarbaldehyde^2^ | Organoheterocyclic compounds | Indoles & derivatives |
| trans-3-Indoleacrylic acid^3a^ | Organoheterocyclic compounds | Indoles & derivatives |
| Serratin^3b^ | Phenylpropanoids & polyketides | Neoflavonoids |
| Hept-4-enedioylcarnitine^3b^ | Lipids & lipid-like molecules | Fatty acyls |
| 2,9-dideoxy-9-glutaramido-2,3-dehydo-N-acetylneuraminic acid^3b^ | Lipids & lipid-like molecules | Fatty acyls |
| DL-α-Aminosuberic acid^3b^ | Organic acids & derivatives | Carboxylic acids & derivatives |
| Indole^3a^ | Organoheterocyclic compounds | Indoles & derivatives |
| Triglochinin^3b^ | Organic oxygen compounds | Organooxygen compounds |
| LMA-P3^3b^ |  |  |
| 9-HpODE^2^ | Lipids & lipid-like molecules | Fatty acyls |
| 12,13-DiHOME^3b^ | Lipids & lipid-like molecules | Fatty acyls |
| N-Acetyl-DL-valine^1^ | Organic acids & derivatives | Carboxylic acids & derivatives |

**Supplementary Fig. 1.** The average relative abundance of bacterial phyla, classes, and genera in the rhizosphere across samples from two bermudagrass genotypes, Tiftuf (TT) and Tifway (TW), and three watering conditions, control (C), medium drought (MD), and severe drought (SD).

**Supplementary Fig. 2**. The average relative abundance of fungal phyla, classes, and genera in the rhizosphere across samples from two bermudagrass genotypes, Tiftuf (TT) and Tifway (TW), and three watering conditions, control (C), medium drought (MD), and severe drought (SD).

**Supplementary Fig. 3.** Mantel test results of Bray-Curtis dissimilarity matrices between root extracts and root exudates, between root exudates and the bacterial species (or annotated genes, i.e., KEGG othology identifiers), and between bacterial species and annotated genes. The Spearman rank correlation method was used to test relationships between those entities, which are represented in boxes. Lines between entities are accompanied with correlation r values and respective FDR values, but the length and thickness of connecting lines are meaningless. False discovery rate (FDR) was used to adjust Spearman rank correlation p values to account for multiple hypothesis testing for reducing false positives.

| A   | B   |
| --- | --- |

**Supplementary Fig. 4.** Part of the whole bipartite networks between grass root exudates (i.e., 1768 peaks) and bacterial species (i.e., 117 with >0.1% relative abundance). For visualization, the subsets of data include the top 18 species with >0.5% relative abundance and the top 50 root exudate compounds with relatively great HPLC peak areas. A and B represent positive and negative connections, respectively, between bacterial species (nodes in green) and root exudates (nodes in blue). The bipartite networks were statistically filtered by Spearman rank correlations to include lines (i.e., edges) connecting significantly (FDR<0.05) between bacterial species and root exudates with r >= 0.7 in A and r <= -0.7 in B. It is worth mentioning that the node size is relevant to the number of edges. False discovery rate (FDR) was used to adjust Spearman rank correlation p values to account for multiple hypothesis testing for reducing false positives.

| A   | B   |
| --- | --- |

**Supplementary Fig. 5.** Part of the whole bipartite networks between grass root exudates (i.e., 1768 peaks) and KEGG orthology identifiers (i.e., 95 K numbers responsible for nutrient transport or uptake, phytohormone regulation, osmolyte production, and scavenging of oxidative species). For visualization, the subsets of data include relatively abundant 18 K numbers and the top 50 root exudate compounds with relatively great HPLC peak areas. A and B represent positive and negative connections, respectively, between annotated genes (nodes in green) and root exudates (nodes in blue). The bipartite networks were statistically filtered by Spearman rank correlations to include lines (i.e., edges) connecting significantly (FDR<0.05) between annotated genes and root exudates with r >= 0.7 in A and r <= -0.7 in B. It is worth mentioning that the node size is relevant to the number of edges. False discovery rate (FDR) was used to adjust Spearman rank correlation p values to account for multiple hypothesis testing for reducing false positives.
